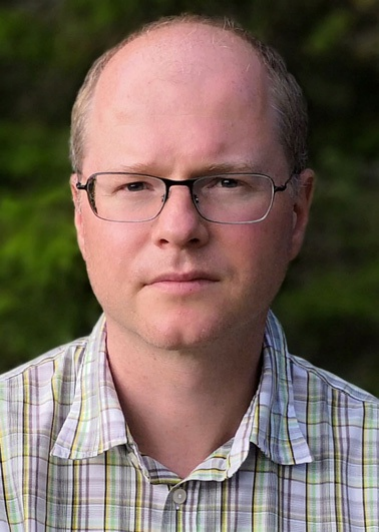# Cationic Organophosphorus Chromophores: A Diamond in the Rough among Ionic Dyes

**DOI:** 10.1002/chem.202001853

**Published:** 2020-10-30

**Authors:** Andrey Belyaev, Pi‐Tai Chou, Igor O. Koshevoy

**Affiliations:** ^1^ Department of Chemistry University of Eastern Finland Yliopistokatu 7 80101 Joensuu Finland; ^2^ Department of Chemistry National (Taiwan) University Taipei 106 Taiwan

**Keywords:** anion–cation interactions, ionic dyes, organophosphorus chromophores, phosphonium salts, phosphorus heterocycles

## Abstract

Tunable electron‐accepting properties of the cationic phosphorus center, its geometry and unique preparative chemistry that allows combining this unit with diversity of π‐conjugated motifs, define the appealing photophysical and electrochemical characteristics of organophosphorus ionic chromophores. This Minireview summarizes the achievements in the synthesis of the π‐extended molecules functionalized with P‐cationic fragments, modulation of their properties by means of structural modification, and emphasizes the important effect of cation‐anion interactions, which can drastically change physical behavior of these two‐component systems.

## Graphical abstract

**Ionic fifteenth**: The diverse synthetic pathways to π‐conjugated systems with quaternary phosphorus, its unique connectivity and tunable electron deficient character, along with the non‐covalent cation‐anion interactions, introduce new photophysical and electrochemical features to ionic chromophores.

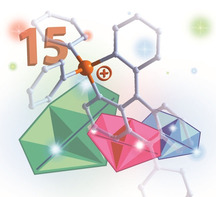

## 1 Introduction

Charged organic molecular chromophores demonstrate remarkably diverse photophysical and electrochemical properties, which have been utilized in a wide range of photonic applications. Due to the ionic nature, many fluorophores (e.g. cyanines, squaraines, rhodamines etc.) have appreciable solubility in protic solvents, and therefore serve as bioimaging agents and chemosensors in physiologic medium.^[1]^ Cationic compounds, which typically incorporate quaternized pyridyl motifs (i.e. viologen‐type species), undergo reversible redox processes and play a key role in the development of electro(fluoro)chromic devices.^[2]^ Furthermore, involvement of charged π‐conjugated molecules in non‐covalent (ion‐π, electrostatic) interactions can substantially affect optical properties of the system and has been actively employed in the design of stimuli‐responsive and smart molecular materials.^[3]^ For instance, intra‐ and intermolecular cation(π^+^)–π stacking comprising pyridinium units has proven to be an efficient approach to enhance fluorescence in solution and in solid, representing a paradigm for switchable luminescence signaling.^[4]^ Ionic dyes, as two‐component salts, are capable of undergoing non‐innocent cation‐anion interactions in aggregated or non‐dissociated (close or contact ion pair) states. Already in the 80′s it was noticed that the formation of ion pairs in the solvents of low polarity influences the absorption spectra of ionic chromophores,^[5]^ pointing to the ground state aggregation. Intuitively, similar association effects might be expected in the excited state, thus modulating the emission characteristics of ionic fluorophores. Despite of this intriguing feature, very limited experimental and theoretical data have been reported in relation to the excited state dynamics of ion pairs during the last decade.^[6]^

The field of ionic dyes has been dominated by nitrogen‐ and oxygen‐containing organic compounds. The heavier pnictogen homologue, phosphorus, has received considerably less systematic attention as positively charged electron deficient center (*λ* ^4^ *σ* ^4^ or *λ* ^4^ *σ* ^3^) within the π‐conjugated chromophore motif, albeit cationic organophosphorus species (mostly phosphonium salts) have long been investigated and applied in organic synthesis, catalysis, and biomedicine.^[7]^ In contrast to P‐cationic photofunctional organic compounds, the P‐neutral congeners (primarily *λ* ^5^ *σ* ^4^ chalcogenide derivatives of tertiary phosphines and P‐heterocycles) have been a subject of thorough investigation,^[8]^ particularly as promising materials for opto‐electronic devices (light‐emitting diodes and solar cells) that was a central topic of a number of detailed reviews.^[9]^

In this respect, herein we mainly focus on the preparation and the properties of the chromophore molecules with the phosphorus‐containing cationic groups attached to or integrated in a π‐conjugated scaffold, where they can act as electron‐accepting components having a distinct impact on the photophysical or electrochemical behavior. Thus, compounds bearing remote phosphonium groups linked via aliphatic spacers (e.g. mitochondrial probes^[10]^), or ionic liquids with non‐chromophoric phosphonium cations^[11]^ are out of the scope of this work.

## 2 Acyclic Organophosphorus Cationic Chromophores

### 2.1 Tailoring terminal phosphonium group to π‐conjugated motifs

**Scheme 1:**
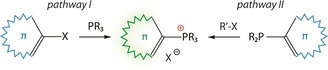
Common approaches to acyclic phosphonium salts.

**Scheme 2:**
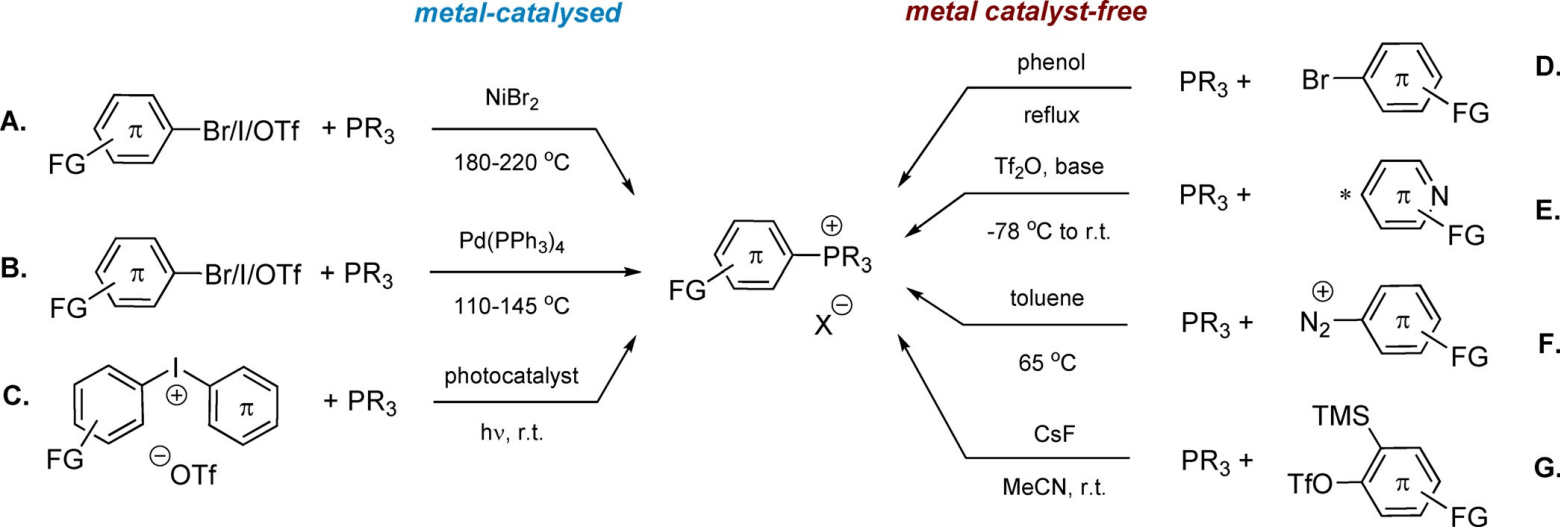
Metal‐catalyzed (A–C) and catalyst‐free (D–G) synthetic routes to phosphonium salts (FG=functional group).

The preparation of phosphonium‐substituted unsaturated organic molecules generally relies on the classical methods of quaternization of tertiary phosphines *λ* ^3^ *σ* ^3^ with alkyl, aryl or alkyne reagents RX, where X is a halide, sulfonate or azide (Scheme 1).^[7]^ The arylation protocol conventionally involves Ni‐ and Pd‐catalyzed P−C bond formation, originally introduced by Horner, Heck and Migita.^[12]^ The efficiency of this process strongly depends on the stereochemistry and electronic properties of the aryl precursor, as well as the reaction conditions (concentration, solvent, catalyst load), which were later optimized by Charette (routes A and B, Scheme 2),^[13]^ and further adapted for polymer synthesis as well.^[14]^ Despite relatively high temperatures (110–145 °C for Pd and 180–220 °C for Ni‐catalyzed P‐C coupling), certain functional groups (alcohols, ketones, aldehydes, amides) tolerate this process.

A mild approach to quaternary P‐arylated compounds is based on photoinduced radical arylation starting from diaryliodonium salts as a radical source in the presence of Ru(bipy)_3_Cl_2_ as a photoredox catalyst (route C, Scheme 2).^[15]^ Interestingly, the use of sterically hindered unsymmetrical Ar‐I^+^‐Mes precursors afforded a range of Ar‐^+^PPh_3_ cations under visible light irradiation (400–410 nm LED) without metal photosensitizer.^[16]^

**Figure 1:**
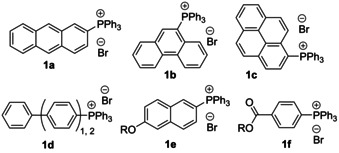
Examples of π‐rich phosphonium salts **1 a**–**f** prepared in refluxing phenol (route D).^[17]^

A facile metal catalyst‐free method to obtain phosphonium salts from oligophenylene and polyaromatic bromides is their coupling with triphenylphosphine in refluxing phenol (e.g. **1 a**–**f**, Figure 1),^[17]^ which allows for the utilization of functionalized precursors containing hydroxyl, ether/ester, carboxyl, and *N*‐unsubstituted indolyl groups.

**Figure 2:**
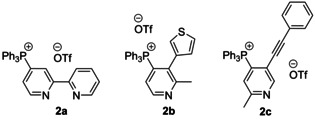
Examples of pyridine phosphonium salts **2 a**–**c**.^[18b]^

A promising regioselective synthesis of 4‐pyridine phosphonium derivatives (**2 a**–**c**, Figure 2) can be carried out under mild conditions by sequential treating the parent pyridine with the triflic anhydride, the phosphine and the base (route E in Scheme 2).^[18]^ Although these salts have been considered as intermediates to functionalize pyridines in the original publications, the described method can also be a facile way to the novel chromophore phosphonium heterocycles. Alternatively, 2‐ and 4‐halopyridines have been used for the arylation of triphenylphosphine.^[19]^

**Figure 3:**
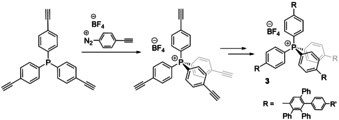
Synthesis of the dendritic phosphonium cations **3**.^[20]^

Aryl diazonium salts are another type of strong electrophiles, which have been used in the synthesis of dendritic phosphonium cations **3** (Figure 3), where a key step of phosphine quaternization carried out at moderate heating (65 °C) tolerates terminal alkyne group (route F).^[20]^

**Scheme 3:**
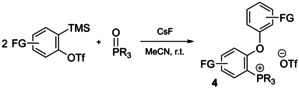
Aryne insertion into the P=O bond to give aryloxy phosphonium triflates **4**.[^17^, ^22^]

In situ generated arynes (e.g. employing (*o*‐trimethylsilyl)aryl triflates and CsF in acetonitrile) also act as efficient agents to quaternize phosphines (route G). This relatively specific method has been applied to prepare a variety of phosphonium triflates, including those with polyaromatic substituents (e.g. **1 b**) and P‐chiral salts.^[21]^ Due to highly electrophilic nature, arynes show a tendency for [2+2] cycloaddition and insertion reactions involving a variety of chemical bonds. Following this reactivity, phosphine oxides were found to be the suitable substrates for the synthesis of phosphonium salts **4** with ethereal substituents (Scheme 3).^[22]^

Phosphine oxides R_3_P=O, readily available compounds, when treated with oxalyl chloride (COCl)_2_ are converted into the chlorophosphonium salts [R_3_PCl]Cl, which consequently react with nucleophilic agents, for example, Grignard reagents R′MgX, to afford quaternized organic derivatives [R_3_PR′]Cl.^[23]^

**Scheme 4:**
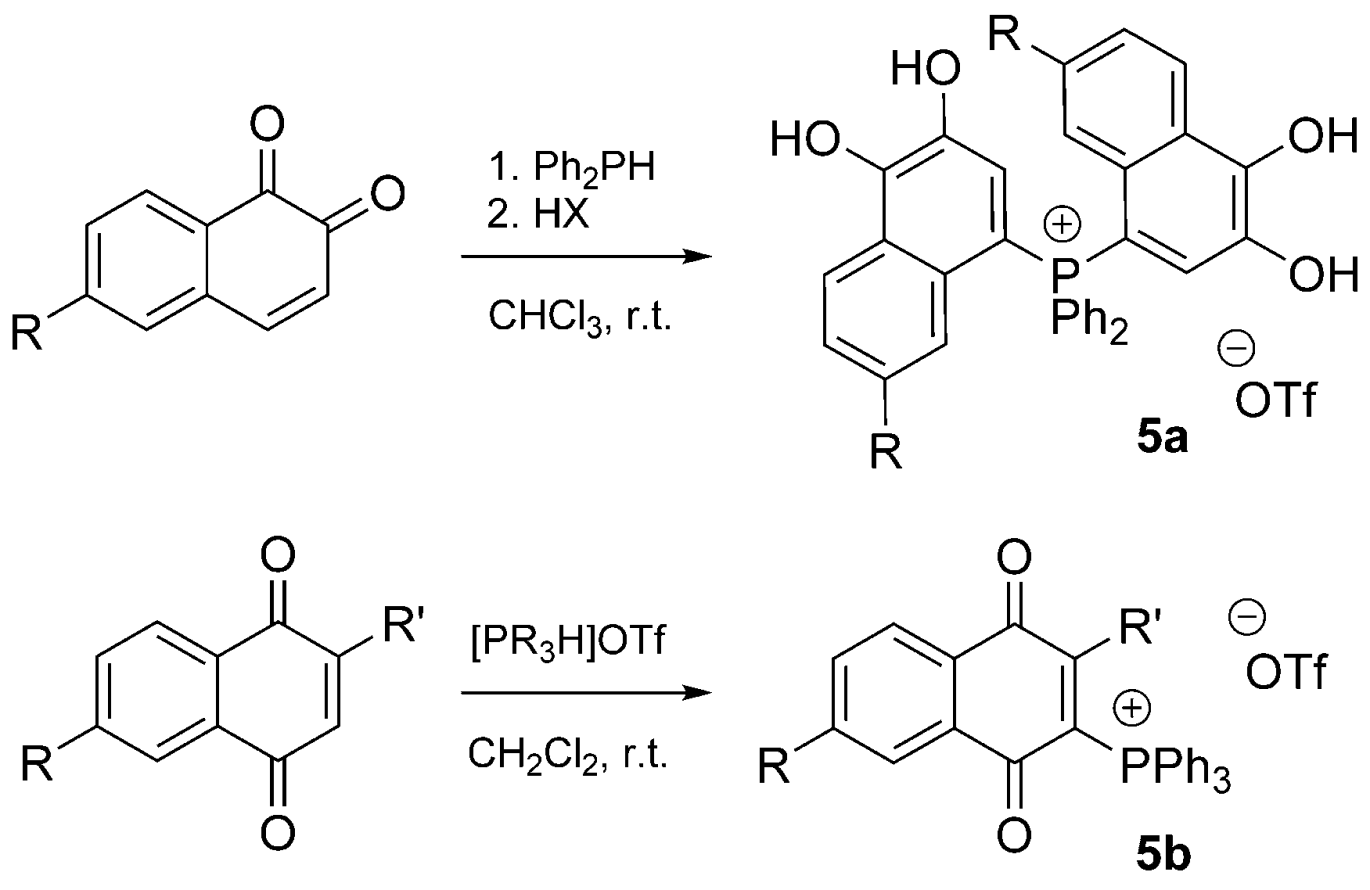
Synthesis of hydroxynaphthyl substituted salts **5 a**,**b**.^[24]^

Reactive P−H bonds, for example, in secondary phosphine PPh_2_H or in protonated tertiary phosphines [R_3_PH]OTf activate 1,2‐ and 1,4‐naphthoquinones, respectively, and afford phosphonium products with dihydroxynaphthyl groups (Scheme 4).^[24]^

**Scheme 5:**
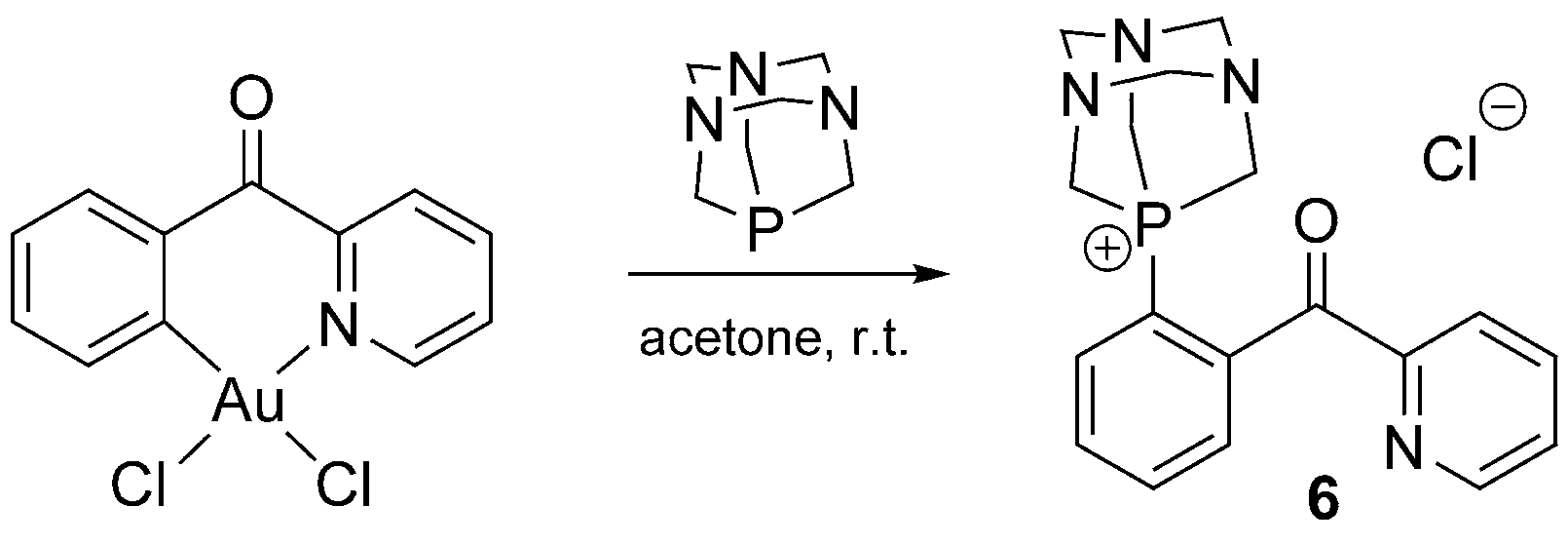
Gold(III)‐mediated synthesis of salt **6** derived from PTA (1,3,5‐triaza‐7‐phosphaadamantane).^[25]^

An intriguing case of gold‐mediated C−P bond formation has been recently disclosed for C^N cyclometalated complex, which interacts with 1,3,5‐triaza‐7‐phosphaadamantane and reductively eliminates phosphonium salt **6** (Scheme 5).^[25]^ Yet not cost effective, this process might illustrate a key step for the future catalyzed C−P coupling reactions.

**Figure 4:**
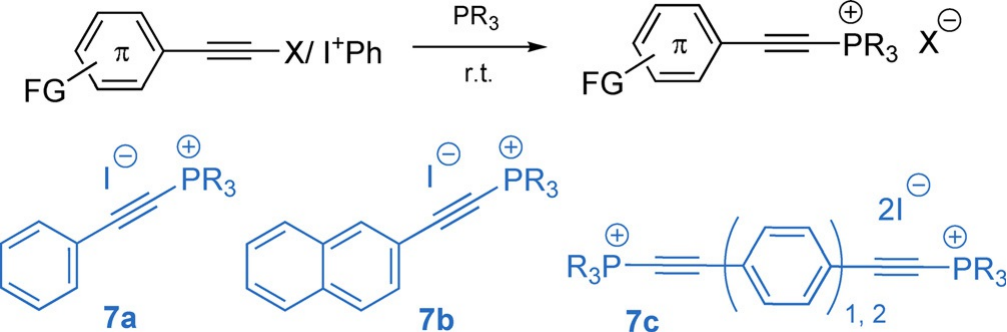
Syntheses of phosphonium salts **7 a**–**c** with alkynyl π‐substituents.[^26^, ^27^]

In addition to phosphonium salts bearing aryl fragments, alkynyl‐containing congeners are conveniently formed upon reacting the tertiary phosphine with electrophilic haloalkynes^[26]^ or (phenyliodonium)alkynes^[27]^ (Figure 4).

Depending on the reactivity and accessibility of the π‐chromophore precursor, the methods belonging to the general pathway I (Scheme 1) might become inefficient, particularly for sterically demanding haloaromatic compounds. As an alternative, such species are first used to obtain tertiary *λ* ^3^ *σ* ^3^ phosphines, which are subsequently quaternized by alkyl halides/triflates (i.e., benzyl‐, naphthyl‐, anthryl bromides, and methyl iodide/triflate) under mild conditions (pathway II, Scheme 1). It should be noted that alkyl phosphonium salts suffer from the lack of stability toward bases (i.e. nucleophilic attack), forming phosphorus ylides.^[7]^

### 2.2 Phosphonium‐containing chromophores

#### 2.2.1 Donor–acceptor systems

The pronounced electron withdrawing character of the pendant *λ* ^4^ *σ* ^4^‐^+^PR_3_ group makes it an attractive unit to tune the energies of frontier orbitals of the chromophore π‐system. In particular, the electron poor nature of ‐^+^PR_3_ substituents can be used for the construction of donor‐acceptor (or push‐pull) ionic compounds with large charge separation, which demonstrate distinct photoinduced intramolecular charge transfer (ICT). The electronic properties of the donor (D), acceptor (A) motifs, and the degree of communication between them by means of a π‐spacer, control the HOMO‐LUMO gap and the ICT, which define the photophysical behavior. In this respect it is essential that the electronic features of the phosphonium motif and the degree of the electron delocalization can be tuned through the modification of R groups. Despite the accessible syntheses described above, such *d*‐π‐A^+^ chromophores are not excessive. The versatile and facile connectivity of the phosphorus atom is also important for the development of multipolar architectures, as it allows for the stepwise synthesis of heterosubstituted compounds of [PR_4−*x*_R'_*x*_]^+^ type.

**Figure 5:**
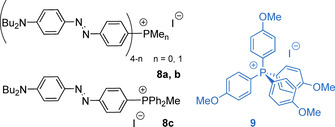
NLO‐active branched octupolar donor‐acceptor cations **8 a**,**b**, **9** and the dipolar congener **8 c**.^[28]^

The 3D octupolar chromophores **8 a**/**b** and **9** (Figure 5) demonstrate non‐linear optical (NLO) behavior in solution and crystalline powder, respectively.^[28]^ Azo‐dyes **8** were expectedly non‐fluorescent. The phosphorus connecting center brings together several virtually non‐interacting chromophore arms that additively increased hyperpolarizability of **8 a** and **8 b** compared to their dipolar relative **8 c**. According to quantum chemical calculations, the phosphonium group ‐^+^PPh_2_Me of **8 c** does not serve as a true electron acceptor. Instead, it strongly polarizes the adjacent phenylene ring and therefore stabilizes the negative charge transfer from the electron rich amino‐phenylene part upon photoexcitation. This ICT results in the inversion of the direction of the dipole moment in the excited state, which is destabilized in more polar solvents leading to the negative solvatochromism (e.g. *λ*_abs_ for **8 a** shift from 511 nm in CHCl_3_ to 499 nm in MeCN). The symmetrical anisole‐derived salt **9** was shown to produce moderate second‐harmonic generation and thus illustrates a way to NLO materials by means of crystal engineering of simple ionic compounds.

**Figure 6:**
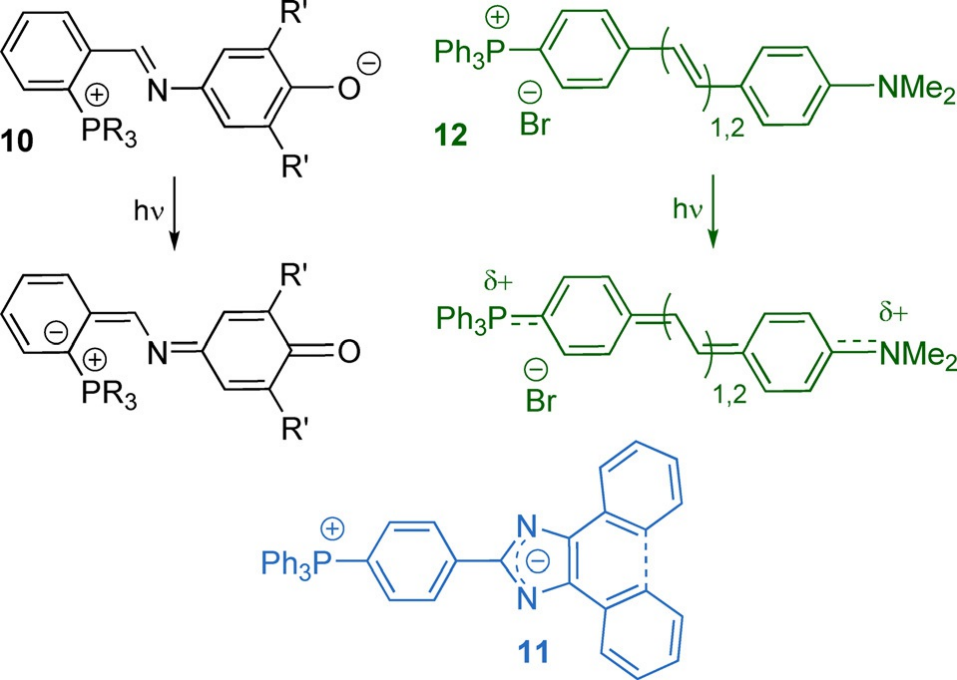
Negatively solvatochromic phosphonium betaine dyes **10**, **11** and the dipolar donor‐acceptor salts **12**; the proposed photoinduced transitions to less polar states for **10** and **12** are shown.^[29]^

The zwitterionic betaine dyes **10** ^[29a]^ and **11** ^[29b]^ (Figure 6) reveal more drastic absorption changes in different solvents than **8**. Thus, **10** (R = R′ = Ph) is red in methanol (*λ*_abs_ = 498 nm), purple in dichloromethane (*λ*_abs_ = 596 nm) and green in toluene (*λ*_abs_ = 686 nm), giving a hypsochromic shift of the lowest energy (charge transfer) absorption band of =5500 cm^−1^ (=188 nm). The substituents at the phosphorus atom in **10** have a distinct influence on the absorption maxima, which appear at 20–30 nm longer wavelengths for R=Ph vs. R=Bu. The alteration of R′ groups (Ph→Bu) shows an opposite effect of approximately the same magnitude; however, the largest hypsochromic shift (ca. 90 nm) of the absorption within this family was achieved when the butyl motif was replaced by the more polar chloride at the R’ positions. Unfortunately, no information was provided concerning the emission properties of compounds **10** and **11**.

The negative solvatochromic behavior of cationic donor‐acceptor salts **12** (*λ*_abs_=422 nm in MeOH and 442 nm dichloromethane for *n=*2) is quite moderate compared to that of betaines **10** and **11**,^[29c]^ which can be attributed to a less polar ground state of the ionic dyes. Compounds **12** were reported to be fluorescent in solution and are evidently prone to photoinduced ICT leading to an appreciable charge redistribution (Figure 6), which in turn might affect the anion‐cation interaction (see section 4 below).

**Figure 7:**
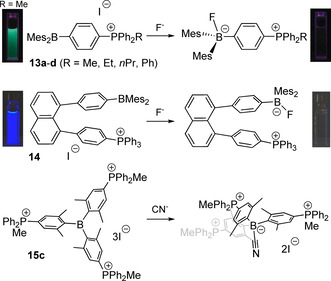
Complexation of the fluoride and cyanide ions with phosphonium boranes **13**,[^30a^, ^30b^] **14** ^[30c]^ and **15 c** ^[32]^ (inset photographs show the corresponding fluorescence responses). Adapted from reference [30a] with permission from The Royal Society of Chemistry (2020). Adapted from reference [30c] with permission from American Chemical Society (2020).

The modulation of the ICT in simple phosphonium salts **13**, **14** containing chemically active arylborane functions suits for highly sensitive fluorescent and colorimetric detection of the fluoride anion (Figure 7).^[30]^ The ionic character of the phosphonium group allows to perform the analysis in aqueous medium. Importantly, the Lewis acidity of the borane unit is readily regulated by the substituents at the P atom and reaches its maximum for compound **13 d** (R=Ph), which shows the highest affinity for the fluoride. This efficient complexation of the F^−^ by the borane combined with cell penetrating ability of the phosphonium group has been lately applied for the selective transport of this anion across phospholipid membrane by **13 b** and similar compounds.^[31]^

Another way to enhance both the acidity of the boron atom and the electrostatic effect for anion sensing is exemplified by the arylboranes (Mes)_3−*x*_B(Ar^P+^)_*x*_ (Ar^P+^=4‐(MePh_2_P^+^)‐2,6‐Me_2_‐C_6_H_2_) bearing one (**15 a**, *x=*1), two (**15 b**, *x=*2) and three (**15 c**, *x=*3, Figure 7) phosphonium substituents.^[32]^ Among these species, the tricationic borane **15 b** is capable of most efficient binding cyanide ions in water at pH of 7 (*K=*1.7×10^7^ m ^−1^), accompanied by clearly detectable changes in the absorption spectra.

**Figure 8:**
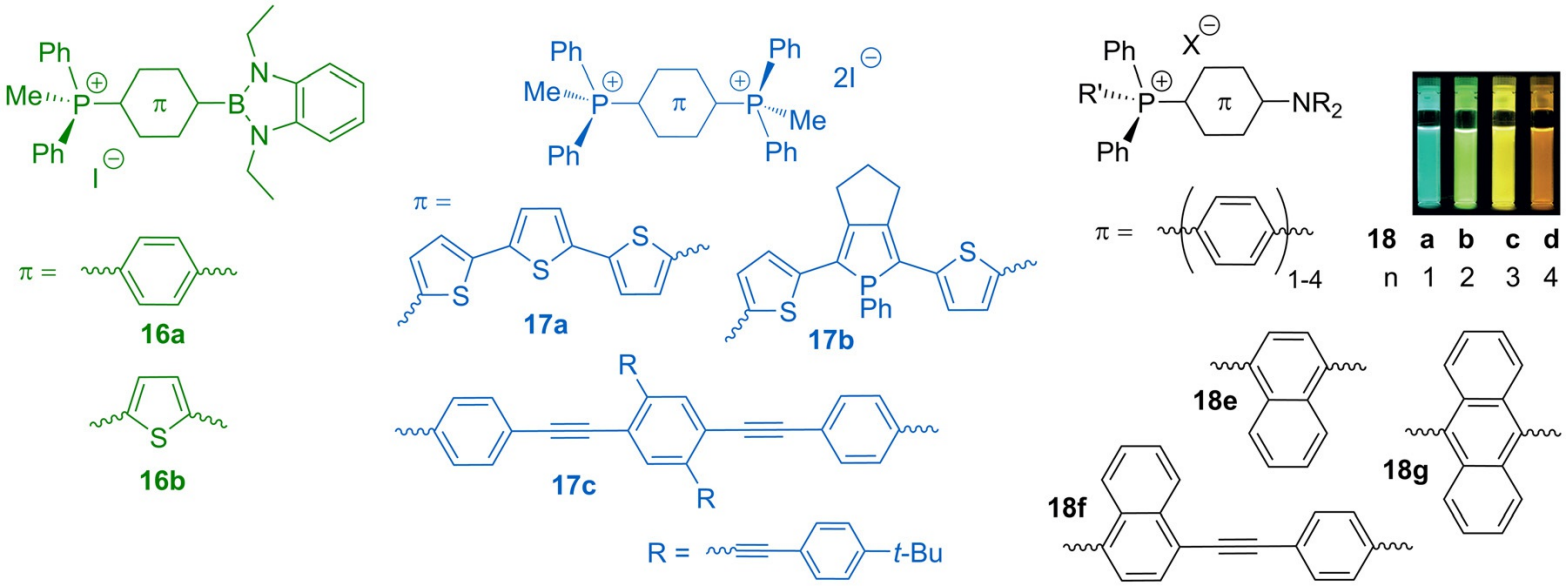
Schematic structures of the *d*‐π‐*A* ^+^ (**16**, **18**)[^30c^, ^36^] and *A* ^+^‐π‐*A* ^+^ (**17**)^[34]^ linear λ^4^σ^4^ phosphonium chromophores (the photo shows dichloromethane solutions of **18 a**–**d** under UV light). Adapted from reference [36] with the permission of Wiley‐VCH GmbH (2020).

Noteworthy, in borane‐phosphonium compounds, the ‐BMes_2_ group, despite being an electron deficient motif, can be the main contributor to HOMO according to the DFT analysis,^[30c]^ and therefore during the photoinduced charge transfer is considered as a weak donor with respect to the cationic highly polarizing ‐^+^PR_3_ fragment. The electron richer 1,3,2‐benzodiazaborole function leads to a more distinct push‐pull character of salts **16** (Figure 8),^[33]^ which reveals visibly smaller HOMO‐LUMO gaps (predicted 3.075–3.099 eV) in comparison to its phosphine‐chalcogenide relatives (predicted 4.093–4.394 eV) due to the lower lying LUMO levels.

The decrease of the optical gap does not necessarily require donor‐acceptor architecture and can be achieved by decorating the π‐system with terminal phosphonium groups; for example, the emission maxima for compounds **17 a**–**c** are bathochromically shifted relatively to their non‐substituted analogues for 33–65 nm (894–3338 cm^−1^).^[34]^ This strategy has been successfully employed in the fabrication of phosphonium‐linked fluorescent polyelectrolytes.^[35]^

Nevertheless, the combination of the phosphonium group with a strong electron donor gives *D*‐π‐*A* ^+^ dyes with wide tunability of the optical band gap (compounds **18**, Figure 8).^[36]^ For example, the oligophenylene‐based salts **18 a**–**d** are brightly fluorescent in dichloromethane solutions (*Φ*_em_=0.71–0.95) with emission wavelength changing from blue (487 nm) to orange (619 nm) upon increasing the number of the phenylene rings in the π‐spacer. Remarkably, the pyridinium donor–acceptor dyes, including the direct congeners of the phosphonium salts **18 a**–**c**, exhibit much lower quantum efficiencies (typically less than 0.01).^[37]^ The use of the polyaromatic (acene) π‐system ultimately results in deep‐red luminescence (*λ*_em_=696 nm for **18 g** in dichloromethane), though with a much lower quantum yield of 0.02 only. These compounds demonstrate good two‐photon absorption (TPA) cross‐section up to 321 GM (Goeppert‐Mayer GM=1×10^−50^ cm^4^ s photon^−1^ molecule^−1^) for phenylene series (**18 d**), while derivative **18 f** reaches the value of 977 GM at the excitation of 800 nm.

#### 2.2.2 Phosphonium‐modified dyes

**Figure 9:**
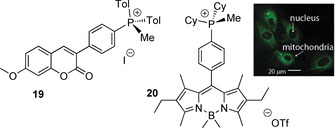
Phosphonium‐modified coumarin (**19**)^[38]^ and BODIPY (**20**)^[39]^ chromophores (inset shows confocal microscopy photo of the H9c2 cell line stained with **20**).

Appending terminal phosphonium motifs to hydrophobic organic dyes is a suitable method to increase their biocompatibility. It has been actively used for the development of various probes to target mitochondria, although in most of the cases the P‐cationic group is electronically innocent as it is isolated from the chromophore scaffold by an aliphatic spacer.^[10]^ The examples of a direct bonding of the phosphonium fragment to the dye molecules are rare, which, for instance, comprise coumarin and BODIPY derivatives **19** ^[38]^ and **20** ^[39]^ (Figure 9), showing specific mitochondrion localization. The quantum yield of **19** reaches 0.91 in dichloromethane (*λ*_em_=441 nm), whereas for unsubstituted 7‐methoxycoumarin the emission intensity is lower (*Φ*=0.53 in buffer and 0.03 in methanol) and the energy is substantially higher (*λ*_em_=394 nm),^[40]^ indicating a non‐innocent effect of ‐^+^PMeTol_2_ electron acceptor on the photophysics of the parent dye.

**Figure 10:**
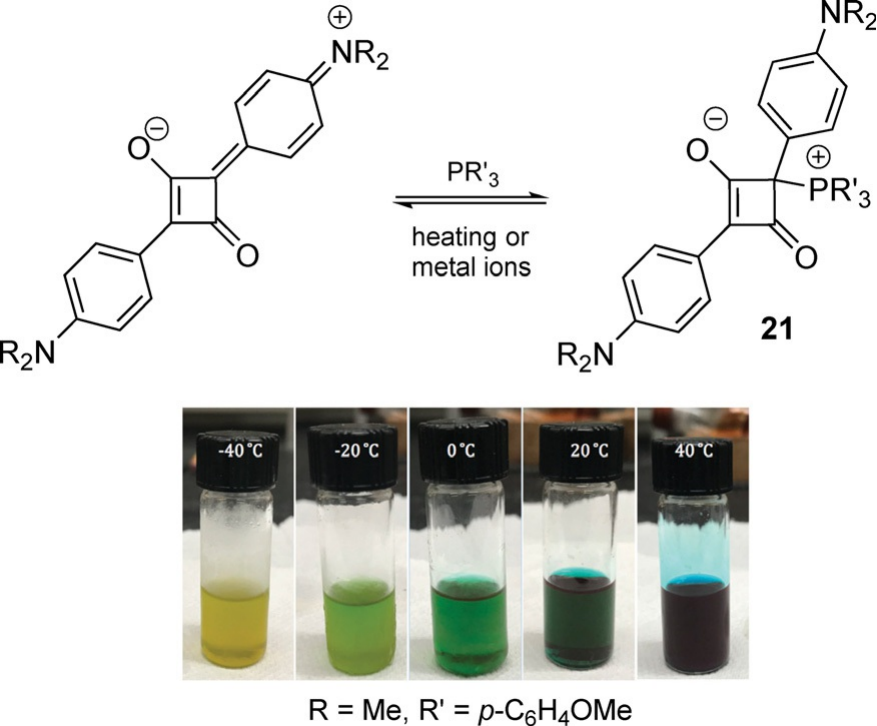
Nucleophilic addition of phosphines to squaraines (photo shows chloroform solutions of the adduct at different temperatures, blue color indicates the recovery of the parent dye upon heating).^[41c]^ Reproduced from reference [41c] with permission from the Royal Society of Chemistry (2020).

The reversible formation of the phosphonium adducts as a result of nucleophilic addition of phosphines to cyanine or squaraine dyes offers unconventional stimuli‐responsive systems.^[41]^ For instance. the equilibrium between intensely blue colored squaraine and the bleached adduct is regulated by nucleophilicity of the PR'_3_ reagent as well as temperature (Figure 10). The adducts **21** also have a potential to serve as chemodosimeters for the metal ions and complexes that have high affinity to phosphine ligands (e.g. Rh, Pd, Ir, Au).^[41c]^

**Figure 11:**
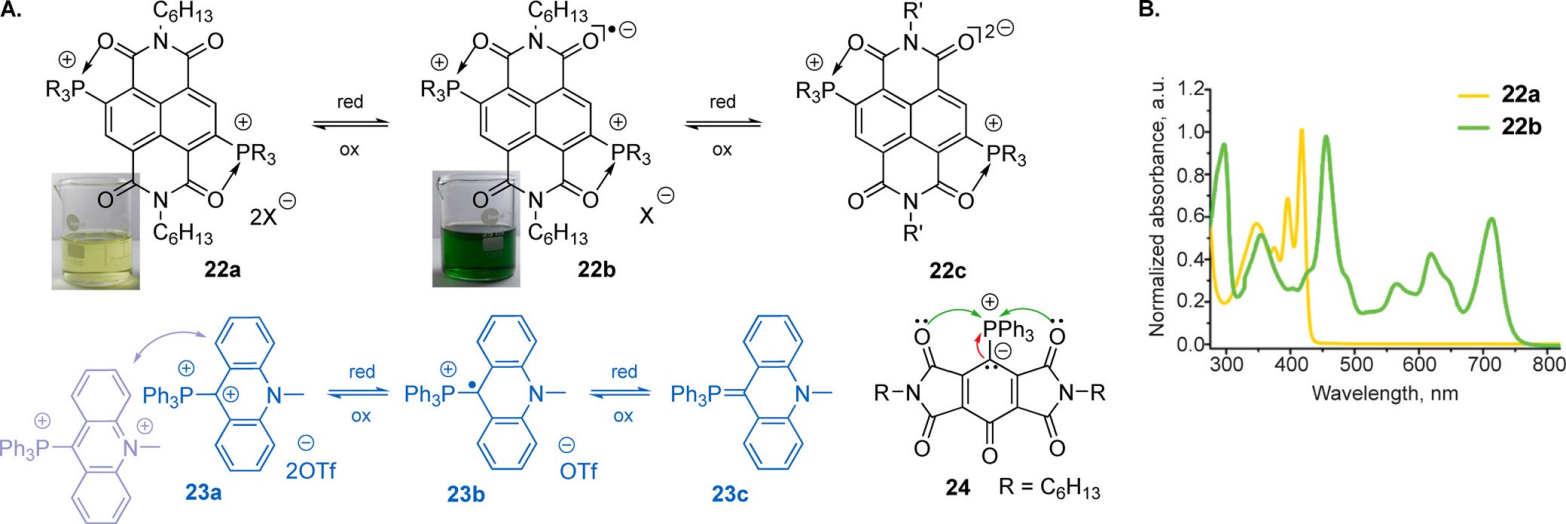
(A) phosphonium dications **22 a** and **23 a** capable of stepwise reduction, and highly stabilized phosphorus ylide diimine **24**; (B) normalized absorption spectra of **22 a** and **22 b** in CHCl_3_ solution.[^42^, ^45^, ^46^, ^47^] Adapted from reference [42] with permission from American Chemical Society (2020).

Decoration of the naphthalenediimide with phosphonium groups generates stable ultra‐electron deficient species **22 a** (Figure 11 A) having exceptionally low LUMO energy of −4.90 eV and the first reduction peak at −0.199 V (vs. Fc^+^/Fc, R=Ph).^[42]^ The corresponding radical ion **22 b**, which can be efficiently prepared from the diimide dibromide and the phosphine under solvent‐free conditions,^[43]^ appears to be highly stable and tolerates conventional workup operations on air. This feature arises to a large extent from hypervalent Lewis acid‐base (^+^P←O) interactions, which are known for other phosphonium salts with hard donors.^[44]^ An easy electron transfer to **22 a** that is accompanied by a dramatic color change (Figure 11 B), and extraordinary stability of radical **22 b** form an attractive platform for switchable electrochromic materials. Moreover, the judicious choice of the R and R' groups made possible the isolation of highly electron rich doubly reduced derivatives **22 c** with diverse color palette.^[45]^

Two step reversible reduction with waves at −0.28 and −0.90 V (vs. Fc^+^/Fc) has been also realized in the α‐phosphonio‐acridinium dication **23 a** that produced air‐stable phosphonio‐radical, and ultimately an easy to decompose antiaromatic phosphorus ylide **23 c** (Figure 11 A).^[46]^ On the other side, the use of pyromellitic diimide motif allows to obtain highly stable chromophoric ylide **24**, which does not show typical Wittig reactivity. Its persistence has been attributed to O:/C: → ^+^P(n_O/C_ → σ*_P–C_) interactions indicated by the natural bond orbital calculations.^[47]^

## 3 Cyclic Organophosphorus Cationic Chromophores

### 3.1 Synthetic approaches to ionic phospha‐annulated systems

**Scheme 6:**
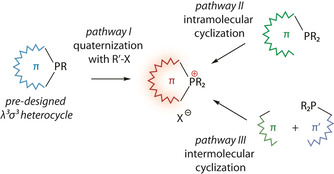
General routes to embed P‐quaternized group into aromatic cyclic scaffold.

The methods of merging the phosphonium function R_2_P^+^< into the π‐conjugated scaffold yet have not been a subject of systematic studies, and therefore the number of general protocols are still quite limited. The reported approaches can be roughly classified into three main categories depicted in Scheme 6.

#### 3.1.1 Quaternization of λ^3^σ^3^ P‐heterocycles

Arguably the most straightforward way to obtain ionic phosphacyclic chromophores is based on the preparation of parent *λ* ^3^ *σ* ^3^‐P cyclic chromophores and their subsequent S_N_2 quaternization (pathway I, Scheme 6), which has been exploited for a wide selection of alkylating agents RX having different leaving groups (X=halides, mesylate, tosylate, or triflate).

**Figure 12:**
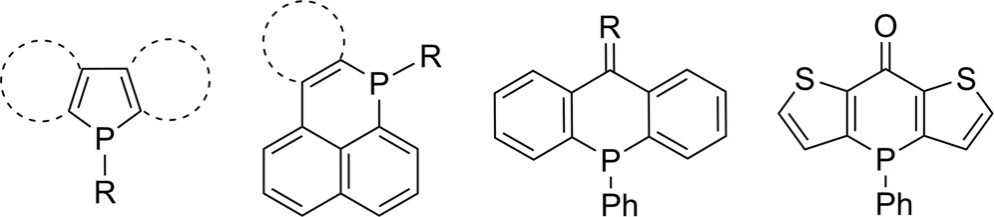
Examples of neutral *λ* ^3^ *σ* ^3^ heterocyclic precursors for P‐quaternization reactions.

Ring‐fused phosphole systems serve as a source for phospholium acenes (Figure 12).^[48]^ The six‐membered organophosphorus cations also can be prepared by alkylation of cyclic *λ* ^3^ *σ* ^3^‐predecessors.^[49]^

In comparison to the tertiary phosphines, lower nucleophilicity of the *λ* ^3^ *σ* ^3^ P atom in phospholes evidently makes it less reactive toward arylation; so far there has been only one example of direct Pd‐catalyzed P‐arylation of the benzo[b]phosphole.^[48g]^

#### 3.1.2 Intramolecular phosphacyclization

**Scheme 7:**
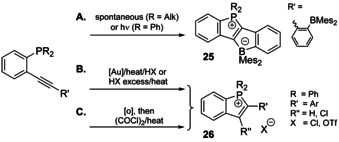
Examples of intramolecular alkyne cyclization reactions to produce phospholium compounds.

Fusing phosphacyclic ionic scaffolds with conjugated hydrocarbon systems can be achieved by means of various intramolecular reactions, which imply the incorporation of P−C bond formation (pathway II, Scheme 6). Following this methodology, the preparation of arylphospholium salts often involves activation of the alkyne motif that is in *ortho*‐position to the phosphorus center (Scheme 7).

An efficient metal‐free synthesis to phospholium‐borate zwitterionic ladder stilbenes **25** was disclosed by Yamaguchi group (route A).^[50]^ The electron‐donating substituents at the phosphorus atoms (R=*t*Bu and Cy) result in immediate nucleophilic cascade cyclization of the intermediate phosphine at room temperature, whereas for R=Ph thermal or photochemical initiation is required.^[51]^

**Scheme 8:**
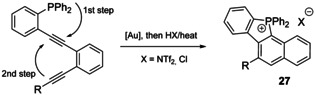
Domino synthesis of fused phospholium derivatives **27**.^[54]^

The closure of 5‐membered phospha‐ring occurs in the course of phosphinoauration of alkynes (route B, Scheme 7), the gold center can be subsequently removed with strong acid to afford cations of a family **26**.^[52]^ This sort of reaction can be carried out without metal reagent as it is promoted by excess of protic acid.^[53]^ The dialkynyl precursors of suitable stereochemistry undergo cascade process, where the first step of stoichiometric phosphinoauration is followed by a gold‐catalyzed cyclization to afford fused π‐extended salts **27** in up to quantitative yields (Scheme 8).^[54]^

Importantly, readily obtainable phosphine oxides can also be transformed into phospholium derivatives in good yields. Their treatment with oxalyl chloride gives electrophilic *λ* ^5^ *σ* ^5^‐chlorophosphonium intermediates, which then transform into the heterocycle at moderately elevated temperature (70 °C, route C, Scheme 7).^[55]^

**Scheme 9:**
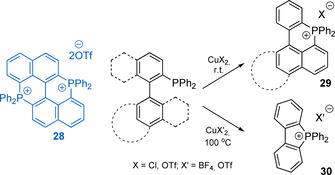
Copper(II)‐mediated phosphacyclization of tertiary phosphines.

Copper(II)‐mediated intramolecular phosphacyclization, based on C−H activation with phosphoniumyl radical formed via single electron transfer, appeared to be a convenient protocol to convert *ortho*‐functionalized tertiary phosphines into five‐ and particularly six‐membered cationic heterocycles (Scheme 9).

The method was first demonstrated for the BINAP diphosphine (2,2′‐bis(diphenylphosphino)‐1,1′‐binaphthyl), which delivered diphosphonium salt **28** in moderate 24 % yield when reacted with 1 equiv. of Cu(OTf)_2_.^[56]^ This strategy was later applied under optimized conditions to a variety of polyaromatic hydrocarbon backbones, affording a series of PAH‐phosphacyclic species **29** (Scheme 9).^[57]^

**Scheme 10:**
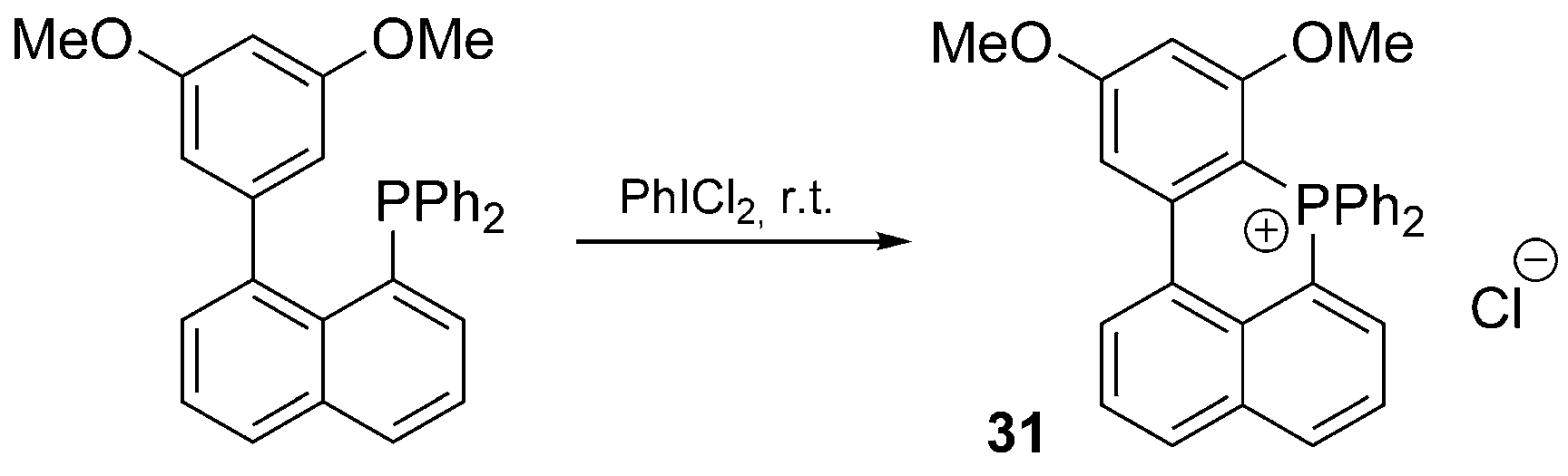
Oxidative synthesis of **31** using PhI^.^Cl_2_.^[59]^

Altering the nature of the *ortho*‐substituent, a similar reaction as well can be employed for the synthesis of phospholium cations **30**.^[58]^ It was also noted that stoichiometric amount of PhI^.^Cl_2_ can oxidize *λ* ^3^ *σ* ^3^ phosphine into a reactive intermediate, which quantitatively cyclizes into phosphaphenalene cation **31** (Scheme 10);^[59]^ however this method was inefficient for the preparation of **29**‐type salts.^[57b]^

#### 3.1.3 Intermolecular phosphacyclization

**Scheme 11:**
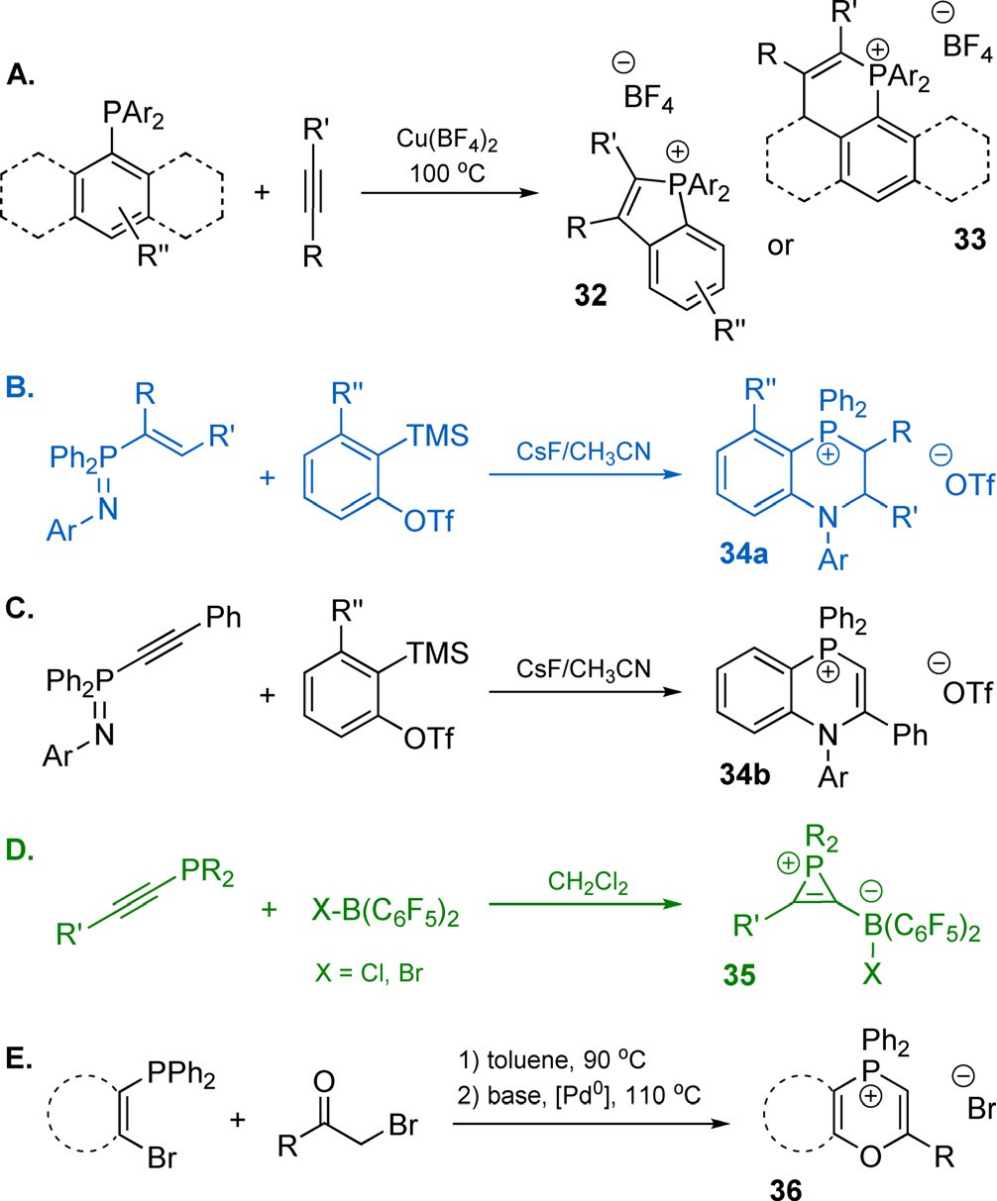
Representative cases of intermolecular cyclization pathways to P‐cationic species.

The intermolecular condensation/cycloaddition reactions leading to the formation of phospha‐rings recently have received considerable attention. An elegant single‐step procedure has been pioneered by Wang and co‐authors, who applied a copper(II)‐mediated cycloaddition of alkynes to tertiary phosphines, yielding five‐ (**32**) or six‐membered (**33**) P‐heterocyclic cations in high yields (Scheme 11 A).^[58]^ The method has been successfully extended by Bouit to PAH‐based diphosphines (naphthalene, pyrene, anthracene, chrysene) with high regioselectivity of bisphosphaacenium cycles.^[60]^

In yet another approach, benzynes undergo insertion into the P=N bond of *P*‐alkenyl/alkynyl‐λ^5^‐phosphazenes. The subsequent cascade intramolecular transformations deliver 1,4‐benzophosphorinium cations **34**, which offer promising possibilities for functionalization and tuning the electronic properties (Scheme 11 B and C).^[61]^ On the other hand, alkynyl phosphines R_2_P‐C≡C‐R' with bulky R groups form the smallest 3‐membered phosphirenium ring upon electrophilic addition of boranes B(Hal)(C_6_F_5_)_2_ (Scheme 11 D).^[62]^ In contrast to the earlier betaine analogs made of B(C_6_F_5_)_3_, zwitterions **35** exhibit high thermal stability, attributed to the halogen substituent of the Lewis acid. The ^+^P,O‐heterocycles **36**, congener to cations **34 b**, are accessible from simple phosphine precursors, which are easily converted into phosphonium ylide intermediates. Subsequent Pd‐catalyzed intramolecular cyclization proceeds as Ullmann‐type O‐arylation giving annulated products with high yields, selectivity, and variable substituents R (Scheme 11 E).^[63]^

### 3.2 Tuning optical properties of ionic phospha‐annulated chromophores

#### 3.2.1 Phospholium‐based dyes

Photophysical behavior of P‐heterocyclic dyes has been predominantly investigated for the systems containing 5‐membered rings, along with a growing number of the reports on the 6‐membered congeners. The electron‐accepting properties of *λ* ^5^ *σ* ^4^‐ and *λ* ^4^ *σ* ^4^‐phosphole derivatives, assigned to the low energy of the LUMO, have been explained by the phenomenon of negative hyperconjugation highlighted in a number of reviews.[^8c^, ^9c^] The electrophilicity of phosphole‐containing species evidently depends on the substituents on the phosphorus atom, and is particularly enhanced for *λ* ^4^ *σ* ^4^ cationic compounds.^[64]^ The incorporation of strongly electron deficient P‐ionic motif into the π‐conjugated scaffold dramatically affects frontier molecular orbitals of the latter. Clearly, the optoelectronic characteristics of such phospha‐annulated systems are primarily dictated by the structure and the composition of the organic framework, but they also can be modulated by means of the ancillary substituents at the phosphorus atom and the counterion component.

**Figure 13:**
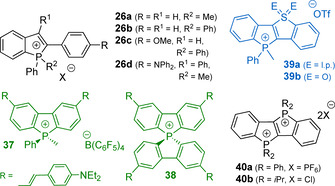
Schematic structures of benzophospholium‐based dyes.

One of the simplest motifs used for the construction of phosphacyclic chromophores is benzophospholium unit (Figure 13). The resulting phenyl‐substituted salts **26 a**–**c** with moderate ICT are intense blue emitters in solution (*λ*_em_=446 nm R=H, 476 nm R=OMe, CH_2_Cl_2_) with quantum yields ranging from 0.75 to 0.89 for PF_6_^−^ or OTf^−^ anions.[^48g^, ^53^] In the case of iodide, the intensity of fluorescence is systematically lower (*Φ*_em_=0.36–0.80) that might be a result of anion‐π charge transfer interaction.^[65]^ The bulkier groups at the phosphorus atom lead to a certain improvement of quantum efficiency by suppressing non‐radiative decay rate; for example, the methylated derivative **26 a** shows twice larger *k_nr_* (3×10^7^ s^−1^) than ethylated and phenylated congeners **26 b**,**c** (*k_nr_*=1.4×10^7^ s^−1^).^[48g]^

The decrease of the optical gap has been also realized in phosphoniafluorenes **37** and **38** by decorating the dibenzophosphospholium motif with diethylamino styryl donors.^[66]^ Salt **38**, a rare example of spiro‐ phosphonium compounds, shows deep‐red to near‐IR fluorescence with *λ*_em_=695 nm (*Φ*_em_=0.1) in toluene and 715 nm in CH_2_Cl_2_ (*Φ*_em_=0.04). The essential impact of the spiro‐structure is reflected not only by the red shift of the absorption and emission bands, but also by almost 3‐fold increase of the TPA cross‐section, which amounts to 1022 GM (at 932 nm) for **38** and 356 GM for **37**.

Modification of the phenyl‐benzophospholium **26 a** with the secondary heteroatom (sulfur) virtually does not change the fluorescence wavelength (**39 a**: *λ*_em_=445 nm, *Φ*_em_=0.36 in CH_2_Cl_2_).^[67]^ The electronic state of sulfur however affects the conjugation within the heterocyclic core, and the electronic behavior of the phosphorus. Oxidation of the S‐atom converts it into an electron accepting group (**39 b**), diminishes the band gap and drastically suppresses the non‐radiative relaxation of the excited state (*k_nr_*=4.3×10^−7^ s^−1^ for **39 a** and 0.1×10^−7^ s^−1^ for **39 b**), giving the quantum efficiency close to unity (**39 b**: *λ*_em_=483 nm, *Φ*_em_=0.99 in CH_2_Cl_2_). Several other thienophospholium derivatives, as well studied by the group of Baumgartner,[^8b^, ^68^] were elegantly engineered via the modification of both the conjugated backbone and the auxiliary substituents at the phosphorus atom. These chromophores demonstrate intriguing stimuli‐responsive fluorochromism and aggregation‐induced emission (AIE), which have been discussed earlier.

The symmetrical dicationic stilbenes **40 a**,**b** (Figure 13) are brightly fluorescent in both solution and solid state.^[69]^ Remarkably, chloride **40 b** gives the quantum yield of 0.74 in water (*λ*_em_=518 nm) with significant Stokes shift of 5780 cm^−1^ that makes this motif an attractive paradigm for bioimaging purposes. On the other hand, relatively low‐lying LUMOs (^CV^ *E*_LUMO_=−4.19 and −4.04 eV for **40 a**,**b**) favor electron injection, suggesting that these electron‐accepting species can be employed for the development of n‐type semiconducting materials.

**Figure 14:**
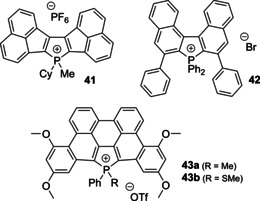
Schematic structures of phospholium dyes polyaromatic cores.

High electron affinity has been also demonstrated by the dyes with extended polyaromatic system. Diacenaphtho phospholium salt **41** (Figure 14) shows low reduction potential of only −1.00 V (vs. Fc^+^/Fc), which is less negative than that of congener neutral phospholes apparently due to the electron deficient nature of the P‐cationic center.^[48b]^

Photoluminescent properties of the phosphacenes are distinctly dictated by the size and stereochemistry of the π‐backbone. If **41** is a weakly deep‐red fluorophore (*λ*_em_=684 nm, *Φ*_em_<0.01 in CH_2_Cl_2_), the binaphthyl derivative **42** has been described as bright green‐yellow emitter in crystalline state.^[70]^

Planarized PAH framework in dibenzophosphapentaphenes **43** provided highly delocalized π MO‐s (Figure 14).^[48c]^ The cationic character of **43 a**,**b** is manifested by the significant contribution of the P atom to the LUMO and higher reduction potential vs. their chalcogenide analogues. The methylated derivative **43 a** reveals moderately intense orange‐red fluorescence (*λ*_em_=599 nm, *Φ*_em_=0.19 in CH_2_Cl_2_). Importantly, air‐ and moisture‐stable thio‐phospholium compound **43 b**, which was obtained by treating the corresponding sulfide with MeOTf, shows spectacular bathochromic shifts of both the lowest energy absorption (from 554 to ca. 610 nm) and emission (to 669 nm) bands compared to **43 a**. Considering the enhanced electron‐accepting ability of R_3_P^+^‐SMe motif, its stability and the easiness of preparation, **43 b** illustrates a very attractive yet undeveloped way to decrease optical band gap of donor‐acceptor organophosphorus chromophores.

**Figure 15:**
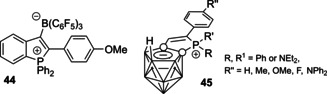
Recent examples of zwitterionic borate‐phospholium fluorophores.[^53^, ^71^]

ICT has been successfully tuned in zwitterionic stilbenes **25** (Scheme 7) and their extended polycyclic derivatives that allowed to vary the emission from green to red‐orange (*λ*_em_=517–623 nm in tetrahydrofuran).[^50^, ^51^] The concept of embedding cationic phospholium (acceptor) and anionic borate (donor) units in the same molecular scaffold was further accomplished in **44**, which was readily obtained from the *ortho*‐alkynyl triphenyl phosphine and B(C_6_F_5_)_3_ as an electrophile in a nearly quantitative yield (Figure 15).^[53]^

The presence of the borate substituent in **44** has a minor influence on the emission parameters (*λ*_em_=471 nm, *Φ*_em_=0.76 in CH_2_Cl_2_) with respect to its predecessor **26 b** (*λ*_em_=476 nm, *Φ*_em_=0.88), but considerably increases the Stokes shift from 4633 cm^−1^ (**26 b**) to 8709 cm^−1^ (**44**).

The series of zwitterionic carboranes **45** are also bright blue emitters (*λ*_em_=446–469 nm, *Φ*_em_=0.34–0.99 in CH_2_Cl_2_).^[71]^ In contrast to the donor‐functionalized **26 d** with red fluorescence,^[48f]^ compound **45** with the same diphenyl‐aniline substituent does not exhibit low energy ICT due to the localization of the HOMO on the carborane fragment. The peculiar feature of compounds **45** lies in their remarkable easiness of the reduction, which occurs at the potential *E*_red_ ranging from −1.02 to just −0.4 V (vs. Fc^+^/Fc) according to electrochemical data.

#### 3.2.2 Chromophores containing six‐membered P‐cationic heterocycles

The development of photofunctional molecular materials based on six‐membered P‐heterocycles has been considerably delayed in comparison to the corresponding phosphole chemistry.^[8d]^ Nevertheless, such phospha‐annulated systems apparently offer rich opportunities for ring modification, and can introduce new chemical and electronic properties, which might be difficult to attain with other cyclic motifs. The so far very limited examples of compounds, incorporating six‐membered P‐cationic rings, are classified as *λ* ^5^ *σ* ^2^‐*endo*,*σ* ^2^‐*exo* species. Their optical behavior is determined by the π‐conjugated backbone, akin to phosphole derivatives. The fluorescence has been detected for compounds with acene backbones, while very weak or no emission was reported for structurally simpler cations (e.g. dibenzophosphonioborine,[^30a^, ^72^] dithienodihydrophosphinine,^[49a]^ acridophosphine^[49c]^ derivatives).

**Figure 16:**
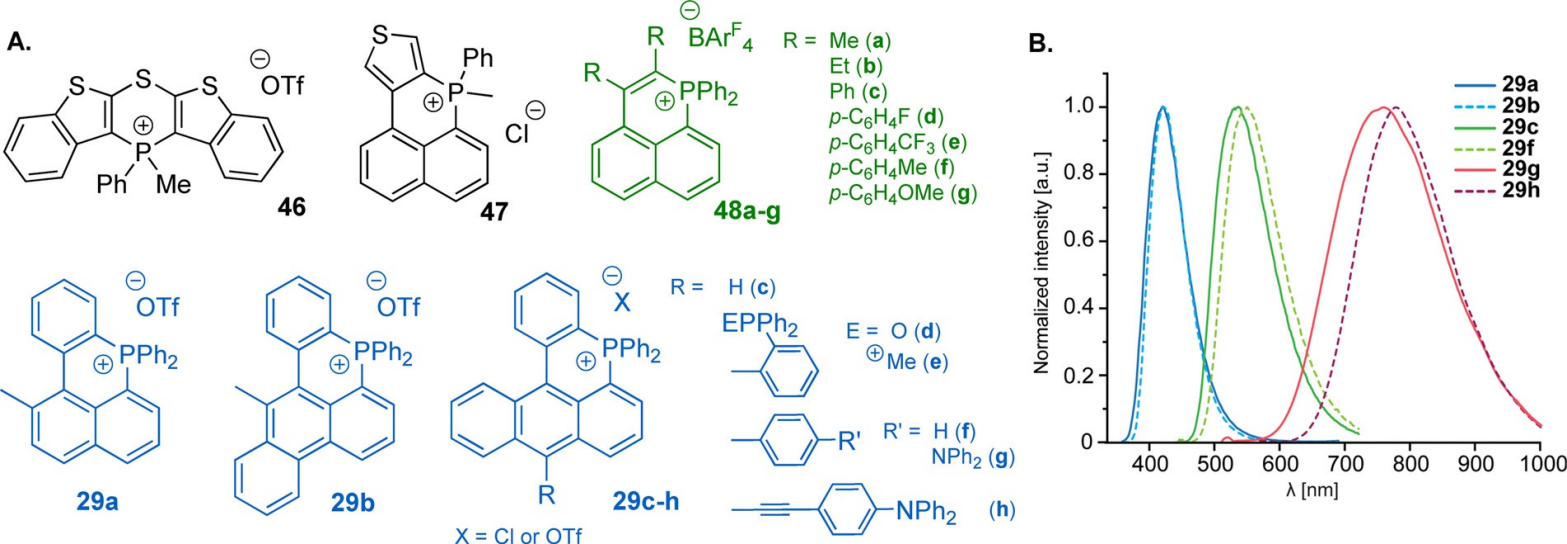
(A) fluorescent monocationic six‐membered phosphacycles fused with acene motifs; (B) emission spectra for selected dyes **29** in CH_2_Cl_2_.

For thiaphosphinine **46** composed of two benzothienyl units (Figure 16), the dynamic luminescence has been observed; the high energy emission band (*λ*_em_=387 nm) transforms into bathochromically shifted band (*λ*_em_=513 nm, *Φ*_em_=0.02) as the concentration increases.^[73]^ This behavior was assigned to an excimer formation, rather unusual for ionic dyes, which also explains solid state fluorescence (*λ*_em_=535 nm).

The naphthalene‐based fluorophores **29 a**, **47** and **48 a**–**g** (Figure 16) reveal strong impact of non‐acene fragment of the backbone on the photophysical performance. Relatively weak deep blue emission of thienyl compound **47** (*λ*_em_=418 nm, *Φ*_em_=0.07 in CH_2_Cl_2_)^[49b]^ is significantly improved in its phenylene congener **29 a** (*λ*_em_=418 nm, *Φ*_em_=0.3 in CH_2_Cl_2_).^[57b]^ Salts **48**, obtained via alkyne insertion into phospharuthenacycles followed by P‐C reductive elimination, are luminescent for aromatic R substituents only.^[74]^ The emission is tuned from *λ*_em_=531 (**48 e**, *Φ*_em_=0.03 in CH_2_Cl_2_) to 588 nm (**48 g**, *Φ*_em_=0.46) primarily by changing the energy of the HOMO, to which the pendant aryl R groups make the dominant contribution, whereas the LUMO localized on the phosphaphenalenium core remains rather unaffected. The modulation of the emission wavelength by moving from R=Ph (**48 c**) to *p*‐C_6_H_4_OMe (**48 g**, Δ*λ*=54 nm, 1720 cm^−1^) is considerably more efficient than that for the analogous structural variation of the abovementioned phospholium dyes **26 b** and **26 c** (Δ*λ*=23 nm, 1067 cm^−1^). It is worth noting that compounds **48 c**–**g** are intensely fluorescent in the solid with quantum yields up to 0.77 (**48 g**) and hypsochromic shift of the emission maxima (*λ*_em_=422–507 nm). These features are attributed to the absence of π‐stacking interactions prevented by the bulky counterion BAr^F^_4_^−^.

The employment of different acene cores together with donor‐acceptor architecture in **29 a**–**h** allows to alter the fluorescence through the entire visible range (*λ*_em_=418–780 nm in CH_2_Cl_2_, Figure 16 B) with good quantum yield even in the near‐IR region (**29 h**, *λ*_em_=780 nm, *Φ*_em_=0.18 in CH_2_Cl_2_).^[57b]^ Due to the ionic nature, most of salts **29** are moderately soluble in water. Importantly, some of these species retain intense emission in aqueous medium (*Φ*_em_=1 for **29 c**–**e** in H_2_O) and demonstrate good TPA properties (the corresponding cross‐section for **29 c**–**f** ranges from 310 to 637 GM measured at 800 nm in water).^[57]^ It has been shown that these photophysical properties, together with high photostability and low toxicity, make anthracene‐based dyes **29 c**–**h** applicable in one‐ and two‐photon cell imaging.

**Figure 17:**
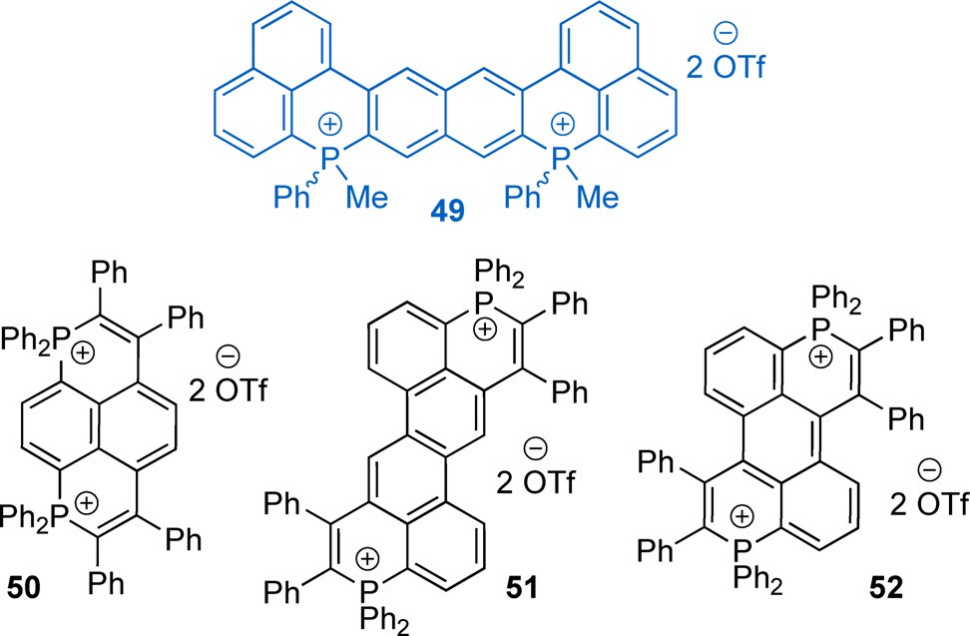
Fluorescent dicationic six‐membered phosphacycles fused with acene motifs.[^49d^, ^60^]

The involvement of the quaternized cycle‐embedded phosphorus atom into the LUMO means that increasing the number of the cationic centers connected via π‐conjugated skeleton necessarily influences frontier orbitals, optical band gap and the electrochemical properties of the system. In line with dicationic nature, the diphosphahexaarene **49** (Figure 17) presents a set of reduction potentials with the lowest value of −1.1 V, while no oxidation was observed.^[49d]^ This points to high stability of the cation, also confirmed by the absence of photodegradation under prolonged irradiation. The vibronic‐structured emission of **49** in solution (*λ*_em_=422 nm, *Φ*_em_=0.8 in CH_2_Cl_2_) remains nearly the same in water (*λ*_em_=423 nm, *Φ*_em_=0.67) providing the opportunities for biovisualization.

An interesting set of dicationic molecules (selected examples **50**–**52** are given in Figure 17) has been recently described by Bouit and co‐workers, prepared from tertiary diphosphines and diphenylacetylene (method A, Scheme 11).^[60]^ Decoration of the naphthalene with phosphininium rings produces 1,8‐bisphosphapyrenium **50** scaffold with orange fluorescence (*λ*_em_=606 nm, *Φ*_em_=0.19 in CH_2_Cl_2_). The extension of the acene framework from naphthalene to chrysene (**51**) however induces blue shift of the emission (*λ*_em_=560 nm, *Φ*_em_=0.06 in CH_2_Cl_2_). Similarly, the binaphthyl dication **28** ^[56]^ reveals further blue shift and emits comparably to **49** (*λ*_em_=453 nm, *Φ*_em_=0.11 in water).^[57b]^ On the downside, **52** possessing anthracene motif shows deep‐red fluorescence (*λ*_em_=672 nm, *Φ*_em_=0.04 in CH_2_Cl_2_) even without involving strong donor groups, which confirms the importance of P^+^‐PAH connectivity but not only the size of the aromatic framework. It is essential that salts **50**–**52** are also bright emitters in solid (*Φ*_em_=0.11–0.39) including **52**, the fluorescence of which is almost unaltered (*λ*_em_=670 nm). Along with the optical data, **52** is the easiest ion to reduce in this series (*E*_red_=−0.64 V vs. Fc^+^/Fc), all dications displaying two separated “viologen‐like” reduction waves in accordance with their structures.

## 4 Cation–Anion Interactions in P‐Cationic Chromophores

**Scheme 12:**
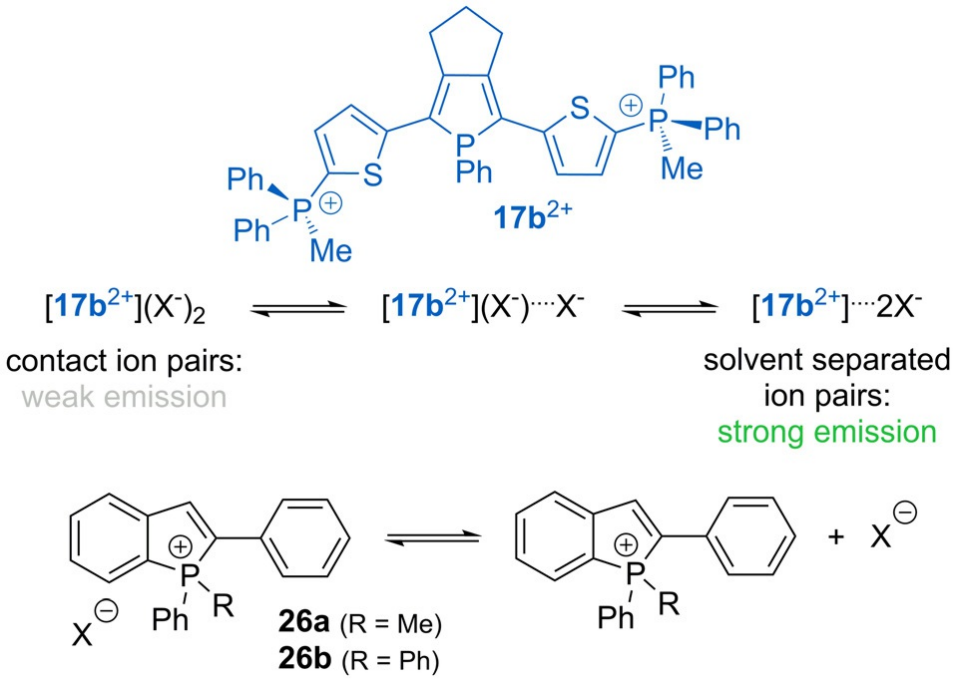
Proposed equilibrium for salts **17 b** and **26 a**,**b** in solution.[^34c^, ^48g^]

**Figure 18:**
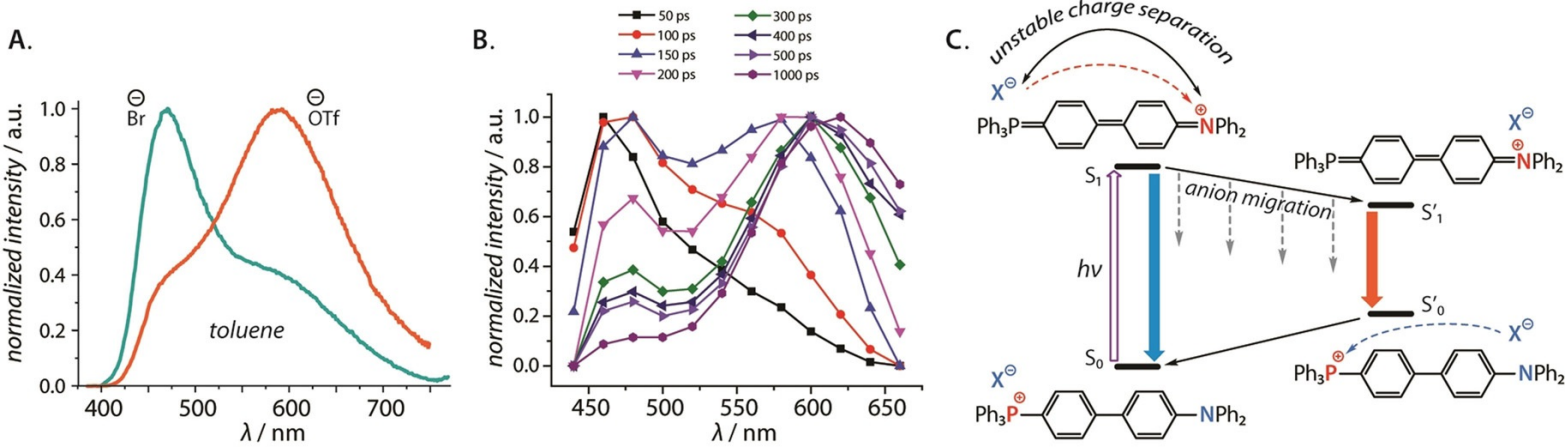
(A) normalized steady state emission spectra of [**18 b** ^+^](Br^−^) and [**18 b** ^+^](OTf^−^); (B) time‐resolved emission spectra of [**18 b**](Br^−^), toluene, 298 K; (C) proposed charge transfer‐induced anion migration resulting in dual emission of **18 b** in non‐polar solvents.^[36]^ Adapted from reference [36] with the permission of Wiley‐VCH GmbH (2020).

The electrostatic noncovalent interactions between the charged components are known to have a substantial effect on the photophysical properties of the ionic dyes.[^6d^, ^6e^, ^6f^] Thus, it has been shown that the intensity of the pyridinium‐π exciplex emission depends on the nature of the counterion, and is enhanced in the presence of PF_6_^−^ but quenched with halides.^[6b]^ The related phenomena of anion‐responsive absorption and/or emission behavior have been also encountered for the P‐cationic chromophores. Thus, the fluorescence of the dicationic compound **17 b** in moderately polar dichloromethane can be regulated by the anion and the concentration (Scheme 12).^[34c]^ At high concentration (which corresponds to the optical density (O.D.) of 1.0) the iodide salt of **17 b** is weakly emissive (*Φ*_em_=0.07), and the fitting of the decay curves gives three different lifetimes (*τ* varies from 0.04 to 2.7 ns). In diluted solution (O.D.=0.1) the quantum yield increases to 0.24, whereas the change of the anion (for PF_6_^−^) or the solvent (e.g., methanol) leads to a single exponential decay (*τ*=2.8 ns) and more dramatic improvement of the luminescence efficiency to 0.86 and 0.77, respectively, at the O.D. of 1.0. These observations were rationalized by the equilibrium between the contact and solvent separated ion pairs. As proposed by the authors, the non‐dissociated state is favored by the iodide and higher concentration in the less polar medium (CH_2_Cl_2_), resulting in (i) the presence of several emissive species, and (ii) quenching of luminescence due to the heavy atom effect. The same group further extended the concept of ion pairs on phospholium fluorophores **26 a,b** (Scheme 12). These species also reveal a systematic increase of the emission intensity in lower polarity solvent for the hexafluorophosphates compared to the iodide analogues, mainly due to the suppression of non‐radiative decay rate.^[48g]^ Although anions have a significant impact on the efficiency of fluorescence for contact ion pairs, the energies of the radiative transitions remain virtually unchanged. In stark contrast, the linear *d*‐π‐*A* ^+^ oligophenylene salts **18 a**–**c** (Figure 8) feature unconventional dual emission in non‐polar solvents (toluene, dioxane, carbon tetrachloride).^[36]^ The ratio of two energetically distinct emission bands (e.g. *λ*_em_=468 and 592 nm for **18 b** in toluene, *Φ*_em_=0.17), which appear as a result of a continuous spectral temporal evolution (Figure 18), strongly depends on the nature of the counteranion, the length of the π‐spacer, solvent viscosity and the temperature. Such relaxation dynamics has been attributed to the hypothesized anion migration that occurs in donor‐acceptor contact ion pairs upon photoinduced ICT in the absence of solvent relaxation (Figure 18 C), and ultimately produces a lower energy emissive excited state. It should be mentioned, in support of the mechanism, that the anion migration in the excited ion pair has been also proposed for some *N*‐heterocyclic (acridinium, naphthoquinolizinium) cationic dyes.[^6a^, ^6d^, ^75^]

**Figure 19:**
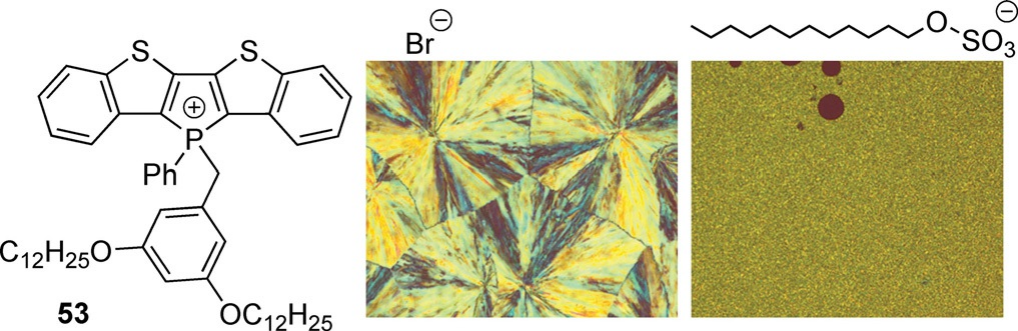
Di(benzothieno)phospholium cation **53** (left), and polarized optical images of **53** salt with Br^−^ and dodecylsulfate anions.^[76]^

No less important can be the role of the counterion in the solid‐state emission of P‐cationic luminophores. It has been noticed for liquid crystalline thienyl phospholium fluorophores that intermolecular arrangement and, as a consequence, the photophysical properties of the bulk material, can be adjusted by steric and electronic effects imposed by counterions.^[48a]^ Hence, in the studied series (Br^−^, BF_4_^−^, BPh_4_^−^, OTf^−^) the anions of larger size diminish the probability of π‐stacking that decreases the difference between the solution and solid state emission, and might increase the quantum yield. Moreover, the substitution of small bromide for amphiphilic dodecylsulfate combined with cationic lipid **53** clearly decreases the crystallinity of the solid and limits the AIE property (Figure 19), highlighting an opportunity to control the morphology and optical characteristics of the ionic material.^[76]^

**Figure 20:**
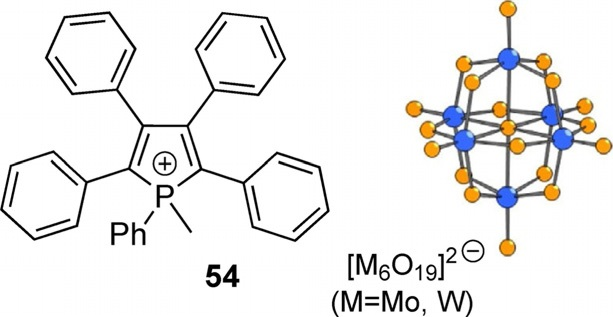
Pentaphenylphospholium **54** coupled with polyoxometalate anion.^[77]^

On the other hand, the anions prone to form strong non‐covalent interactions are capable to facilitate AIE behavior. AIE‐active phospholium **54** crystallized with polyoxometalates [M_6_O_19_]^2−^ (M=Mo, W), shown in Figure 20, are involved in the extensive network of anion‐π interactions and C−H⋅⋅⋅O hydrogen bonding, while the π–π stacking is prevented upon incorporating the bulky anions as spacers. These multiple contacts rigidify the fluorescent cation that raises the quantum yield almost 3‐fold (*Φ*_em_=0.43) with respect to its triflate salt (*Φ*_em_=0.15) and ca. 90‐fold compared to that in the solution.^[77]^

**Figure 21:**
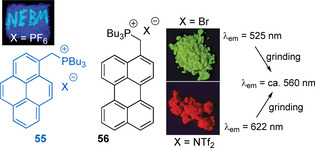
Phosphonium salts **55** and **56** containing polyaromatic chromophores (photos show the effect of grinding for **55** (left), and of the counterion on the solid state emission of **56** (right).^[78]^ Adapted from reference [78] with permission from The Royal Society of Chemistry (2020).

Although phosphonium groups in salts **55** and **56** are separated from the polyaromatic π‐systems by the methylene spacer (Figure 21), the non‐innocent interactions between cations and anions give rise to interesting mechanofluorochromic properties.^[78]^ In particular, variation of counterions from Br^−^ to NTf_2_^−^ for **56** changes the emission of crystalline materials from green (525 nm) to red‐orange (622 nm). Upon grinding, salts [**56** ^+^](X^−^) exhibit nearly identical fluorescence (ca. 560 nm) due to the formation of amorphous phase, that is, the direction of mechanochromic shift of the emission is triggered from red (Br^−^) to blue (NTf_2_^−^) simply by means of the anion.

The anion‐cation interactions (anion=Cl^−^, Br^−^, I^−^, NO_3_^−^, BF_4_^−^, PF_6_^−^, BPh_4_^−^) involving phosphonio‐diimide radical ions of the type **22 b** (Figure 11 A) as well have shown an ability to amend the photophysical and magnetic properties of the cationic π‐radical systems. In crystals, the radical cations form various supramolecular assemblies utilizing charge‐assisted hydrogen bonding and anion‐π contacts. Ion pairing effect appears to be non‐innocent in solution too, especially in the solvent of lower polarity. For example, the UV‐vis measurements supported by theoretical calculations suggest that nitrate radical salts undergo unusual “reverse” π→anion photoinduced electron transfer in contrast to other anions.^[79]^

## 5 Concluding Remarks

In this minireview we tried to highlight the reported data on the organophosphorus ionic chromophores, in which the P‐cationic groups play an active role in composing the electronic structures, and therefore show a clear influence on the optical and/or electrochemical properties. These dyes still can be considered as an emerging class of compounds as most of the relevant works have been published during the last decade. The diverse synthetic pathways to π‐conjugated P‐cations, particularly to P‐heterocyclic species, go far beyond simple quaternization of *λ* ^3^ *σ* ^3^ tertiary phosphines, and can produce intriguing molecular scaffolds, many of which very probably are still undisclosed. Strong electron deficient character of *λ* ^4^ *σ* ^4^ (and *λ* ^4^ *σ* ^3^) phosphorus centers, which can be tuned with the help of the substituents, is immensely useful in the construction of fascinating donor‐acceptor systems exhibiting versatile charge transfer and redox behavior. In addition, the unique connectivity of the pyramidal phosphorus atom allows the design and accessible preparation of branched multipolar and multichromophore architectures, although this option has been exploited yet to a very limited extent. Taking into account the charged nature of the described chromophores, the effect of cation‐anion interactions and ion pair formation both in the solid and in solution cannot be neglected. Albeit ion pairing brings an additional degree of freedom, which is often difficult to evaluate precisely and to control in reproducible and predictable manner, simultaneously it can greatly diversify physical and chemical properties of two‐component aggregates, leading to unconventional phenomena and functionalities that may be far‐reaching in both fundamental and applications.

## Conflict of interest

The authors declare no conflict of interest.

## Biographical Information

*Dr. A. Belyaev received his M.Sc. in inorganic chemistry with Prof. S. P. Tunik from Saint‐Petersburg State University (Russia)*, *and Ph.D. degree in organometallic chemistry under the supervision of Prof. I. O. Koshevoy (University of Eastern Finland) in 2020. Currently*, *he is a post‐doctoral researcher in the group of Prof. Andreas Steffen*, *Technical University of Dortmund (Germany). His research interests encompass the design*, *synthesis and the photophysics of organometallic and inorganic luminescent molecular materials*.

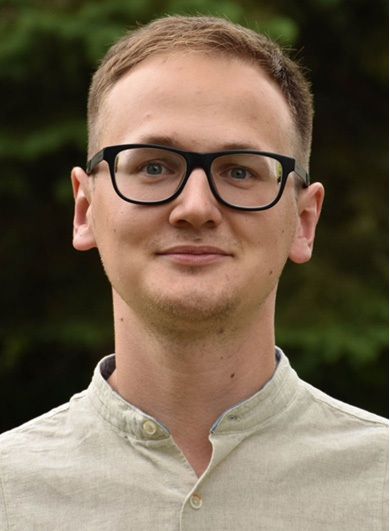

## Biographical Information

*Prof. Pi‐Tai Chou earned his Ph.D. in Chemistry and Biochemistry from the Florida State University. He was a postdoctoral fellow in the University of California at Berkeley. He is currently a distinguished chair professor of Chemistry department and director of Center for Emerging Material and Advanced Devices in National Taiwan University (Taiwan). Prof. Chou is an expert in molecular spectroscopy and dynamics. He is also involved in the fundamentals and applications of solar cell*, *organic light emitting diodes*, *energy storage*, *bio‐sensing and bio‐imaging*.

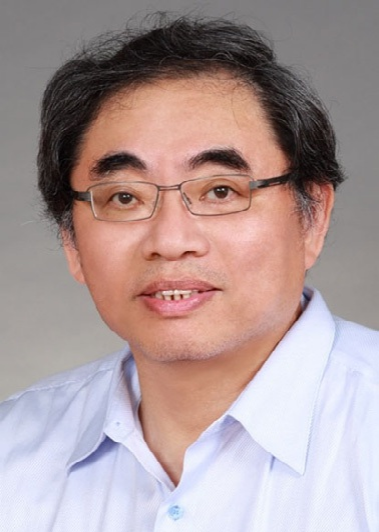

## Biographical Information

*Prof. Igor Koshevoy obtained his M.Sc. and Ph.D. degrees with Prof. S. P. Tunik from St.‐Petersburg State University (Russia)*, *in 1999 and 2002. After post‐doctoral stays at the Universities of Toronto (Canada)*, *Valencia (Spain)*, *and the University of Eastern Finland*, *he was appointed as an Associate Professor and later a Full Professor at the University of Eastern Finland. His work is focused on synthetic inorganic and organometallic chemistry of late transition metals*, *non‐covalent interactions*, *with an emphasis on producing light‐emitting molecular materials*.